# Special Issue “Dietary (Poly)Phenols and Health”

**DOI:** 10.3390/nu14071402

**Published:** 2022-03-28

**Authors:** Přemysl Mladěnka

**Affiliations:** Department of Pharmacology and Toxicology, Faculty of Pharmacy in Hradec Králové, Charles University, Ak. Heyrovského 1203, 50005 Hradec Králové, Czech Republic; mladenkap@faf.cuni.cz

Interest in understanding the mechanisms of the positive effects of dietary phenolic and polyphenolic compounds on human health has markedly increased in recent years. In particular, there is a clear rise of investigations related to metabolic aspects of these compounds. Specifically, a very steep increase is obvious in the case of flavonoids (Figure 1). The traditional testing of dietary compounds using in vitro models without knowing their pharmacokinetics and hence the real biological exposure to those compounds and their metabolites, has been receding, since it might give the readers misleading conclusions about the real clinical impact of these compounds. Indeed, several strong reviews have critically emphasized low bioavailability of parent dietary phenolic substances [1,2,3,4]. On the other hand, it should be mentioned, that the unabsorbable part of phenolics is metabolized by human microbiota and gives rise to a row of simpler phenolic compounds that are more bioavailable and thus could be responsible for the final effect on human organisms [5,6,7,8].

I am pleased that 19 papers published in this Special Issue [9,10,11,12,13,14,15,16,17,18,19,20,21,22,23,24,25,26,27], present novel findings and adhere to the above-mentioned current trends. I personally consider the fact, that some papers in this issue also reported negative or only mild effects of the tested compounds to be very important as well. Notwithstanding that this likely represents a blind alley, it is also very important information for future research directions.

The papers in this issue also covered different experimental and scientific approaches. There are seven review papers, one meta-analysis, one epidemiological study, four clinical trials, three animal experiments, and three in vitro studies. The readers can find the following interesting information in this Special Issue: Sova and Saso’s paper comprehensibly summarizes the current data on different biological aspects of hydroxycinnamic acids with an emphasis on bioavailability, metabolism, and possible pharmacological effects [22]. Another paper sums up the current knowledge on oil palm phenolics, which is in fact an aqueous waste produced during the milling process that can be a potential source of biologically active phenolic compounds [12]. The impact of polyphenols on stroke from both animal experimentation and human trials is summarized in other paper [20]. Yessenkyzy et al. convey an interesting and relatively novel concept of polyphenols as compounds able to induce autophagy, which is similar to caloric restriction and seems to have an impact on humans, in particular at an advanced age. The mechanism(s) of this process are also discussed by the authors of [27]. Current knowledge on sakuranetin, one of less explored flavanones, was also published in this issue [23]. Wüpper et al. summarize available data on the chemical composition, pharmacological effects, and safety of Kuding tea, which contains polyphenolic compounds and triterpenic saponins [26]. A review on the composition and pharmacological activity of garlic mentions that flavonoids are also present in garlic [10].

Human interventional studies published in this Special Issue included: (a) a study that found that riceberry rice bread rich in anthocyanins caused a significantly lower postprandial glucose peak in comparison to Hom Mali bread [11] and similarly, a polyphenol-rich functional food breakfast caused a lower glucose peak than ready-to-eat breakfast cereal [14]; (b) a comparison of the effect of beverages enriched with phenol-rich extracts from apple, blueberry, and coffee berry on cognition and mood [13]; (c) a study that found that Sakurajima radish administration did not reduce arterial blood pressure but showed a tendency to decrease heart rate [21]. An epidemiological study suggested, that intake of two groups of polyphenols (stilbenes and flavonoids) was associated with lower obesity prevalence. Additionally, the impact of these groups on the human microbiome was reported [18]. A meta-analysis of clinical trials related to supplementation with polyphenols reported a moderate effect on psychomotor function. This influence seemed to be more probable in young and middle-aged adults, and the potential impact of bioavailability was also analyzed [9].

Data from animal studies have shown: (a) combinations of colonic metabolites of quercetin can have a more pronounced effect on arterial blood pressure in rats [19]; (b) the flavonol robinin only mildly improved the effect of methotrexate in an adjuvant-induced arthritis model in rats [24]; (c) the impact of a polyphenol-rich extract from *Aronia melanocarpa* (chokeberries) on liver collagen, matrix metalloproteinases, and their tissue inhibitors in rats chronically exposed to cadmium [15].

Two papers analyzed metabolic issues in vitro. Mohos et al. reported the important interactions of quercetin and its conjugates with several transporters, some of which were found even in sub-micromolar concentrations and with cytochrome P450 isoenzymes [17]. Lněničková et al. documented that some prenylflavonoids are able to mildly modulate mRNA expression and activity of drug-metabolizing enzymes even at a concentration of 1 µM [16]. The final paper in this Special Issue reported interactions of flavonolignans from silymarin and their sulfate conjugates with zinc ions as well as with zinc-containing alcohol dehydrogenase [25].

## Figures and Tables

**Figure 1:**
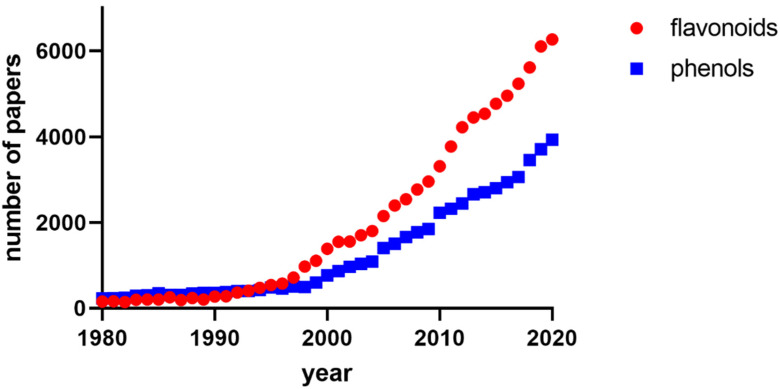
Increases in the number of papers investigating metabolites or metabolism of flavonoids and phenols. PubMed was used for the analysis. Keywords employed were ‘flavonoid and metabol* AND (“X”[Date-Publication]: “X”[Date-Publication])‘ or ‘phenol* and metabol* AND (“X”[Date-Publication]: “X”[Date-Publication])‘.

## References

[1] Del Rio D., Rodriguez-Mateos A., Spencer J.P., Tognolini M., Borges G., Crozier A. (2013). Dietary (poly)phenolics in human health: Structures, bioavailability, and evidence of protective effects against chronic diseases. Antioxid. Redox. Signal..

[2] Di Lorenzo C., Colombo F., Biella S., Stockley C., Restani P. (2021). Polyphenols and Human Health: The Role of Bioavailability. Nutrients.

[3] Najmanova I., Voprsalova M., Saso L., Mladenka P. (2020). The pharmacokinetics of flavanones. Crit. Rev. Food Sci. Nutr..

[4] Williamson G., Kay C.D., Crozier A. (2018). The Bioavailability, Transport, and Bioactivity of Dietary Flavonoids: A Review from a Historical Perspective. Compr. Rev. Food Sci. Food Saf..

[5] Llorach R., Garrido I., Monagas M., Urpi-Sarda M., Tulipani S., Bartolome B., Andres-Lacueva C. (2010). Metabolomics study of human urinary metabolome modifications after intake of almond (*Prunus dulcis* (Mill.) D.A. Webb) skin polyphenols. J. Proteome Res..

[6] Pimpao R.C., Ventura M.R., Ferreira R.B., Williamson G., Santos C.N. (2015). Phenolic sulfates as new and highly abundant metabolites in human plasma after ingestion of a mixed berry fruit puree. Br. J. Nutr..

[7] Rodriguez-Mateos A., Rendeiro C., Bergillos-Meca T., Tabatabaee S., George T.W., Heiss C., Spencer J.P. (2013). Intake and time dependence of blueberry flavonoid-induced improvements in vascular function: A randomized, controlled, double-blind, crossover intervention study with mechanistic insights into biological activity. Am. J. Clin. Nutr..

[8] Feliciano R.P., Boeres A., Massacessi L., Istas G., Ventura M.R., Nunes Dos Santos C., Heiss C., Rodriguez-Mateos A. (2016). Identification and quantification of novel cranberry-derived plasma and urinary (poly)phenols. Arch. Biochem. Biophys..

[9] Ammar A., Trabelsi K., Boukhris O., Bouaziz B., Müller P., Glenn J.M., Chamari K., Müller N., Chtourou H., Driss T. (2020). Moderators of the Impact of (Poly)Phenols Interventions on Psychomotor Functions and BDNF: Insights from Subgroup Analysis and Meta-Regression. Nutrients.

[10] El-Saber Batiha G., Magdy Beshbishy A., Wasef G.L., Elewa Y.H.A., A. Al-Sagan A., Abd El-Hack M.E., Taha A.E., M. Abd-Elhakim Y., Prasad Devkota H. (2020). Chemical Constituents and Pharmacological Activities of Garlic (*Allium sativum* L.): A Review. Nutrients.

[11] Chusak C., Pasukamonset P., Chantarasinlapin P., Adisakwattana S. (2020). Postprandial Glycemia, Insulinemia, and Antioxidant Status in Healthy Subjects after Ingestion of Bread made from Anthocyanin-Rich Riceberry Rice. Nutrients.

[12] Ibrahim N.I., Fairus S., Naina Mohamed I. (2020). The Effects and Potential Mechanism of Oil Palm Phenolics in Cardiovascular Health: A Review on Current Evidence. Nutrients.

[13] Jackson P.A., Wightman E.L., Veasey R., Forster J., Khan J., Saunders C., Mitchell S., Haskell-Ramsay C.F., Kennedy D.O. (2020). A Randomized, Crossover Study of the Acute Cognitive and Cerebral Blood Flow Effects of Phenolic, Nitrate and Botanical Beverages in Young, Healthy Humans. Nutrients.

[14] Kennedy S.J., Ryan L., Clegg M.E. (2020). The Effects of a Functional Food Breakfast on Gluco-Regulation, Cognitive Performance, Mood, and Satiety in Adults. Nutrients.

[15] Kozłowska M., Brzóska M.M., Rogalska J., Galicka A. (2020). The Impact of a Polyphenol-Rich Extract from the Berries of Aronia melanocarpa L. on Collagen Metabolism in the Liver: A Study in an In Vivo Model of Human Environmental Exposure to Cadmium. Nutrients.

[16] Lněničková K., Šadibolová M., Matoušková P., Szotáková B., Skálová L., Boušová I. (2020). The Modulation of Phase II Drug-Metabolizing Enzymes in Proliferating and Differentiated CaCo-2 Cells by Hop-Derived Prenylflavonoids. Nutrients.

[17] Mohos V., Fliszár-Nyúl E., Ungvári O., Kuffa K., Needs P.W., Kroon P.A., Telbisz Á., Özvegy-Laczka C., Poór M. (2020). Inhibitory Effects of Quercetin and Its Main Methyl, Sulfate, and Glucuronic Acid Conjugates on Cytochrome P450 Enzymes, and on OATP, BCRP and MRP2 Transporters. Nutrients.

[18] Mompeo O., Spector T.D., Matey Hernandez M., Le Roy C., Istas G., Le Sayec M., Mangino M., Jennings A., Rodriguez-Mateos A., Valdes A.M. (2020). Consumption of Stilbenes and Flavonoids is Linked to Reduced Risk of Obesity Independently of Fiber Intake. Nutrients.

[19] Najmanová I., Pourová J., Mladěnka P. (2020). A Mixture of Phenolic Metabolites of Quercetin Can Decrease Elevated Blood Pressure of Spontaneously Hypertensive Rats Even in Low Doses. Nutrients.

[20] Parrella E., Gussago C., Porrini V., Benarese M., Pizzi M. (2021). From Preclinical Stroke Models to Humans: Polyphenols in the Prevention and Treatment of Stroke. Nutrients.

[21] Sasaki M., Nonoshita Y., Kajiya T., Atsuchi N., Kido M., Chu D.-C., Juneja L.R., Minami Y., Kajiya K. (2020). Characteristic Analysis of Trigonelline Contained in Raphanus sativus Cv. Sakurajima Daikon and Results from the First Trial Examining Its Vasodilator Properties in Humans. Nutrients.

[22] Sova M., Saso L. (2020). Natural Sources, Pharmacokinetics, Biological Activities and Health Benefits of Hydroxycinnamic Acids and Their Metabolites. Nutrients.

[23] Stompor M. (2020). A Review on Sources and Pharmacological Aspects of Sakuranetin. Nutrients.

[24] Tsiklauri L., Švík K., Chrastina M., Poništ S., Dráfi F., Slovák L., Alania M., Kemertelidze E., Bauerova K. (2021). Bioflavonoid Robinin from Astragalus falcatus Lam. Mildly Improves the Effect of Metothrexate in Rats with Adjuvant Arthritis. Nutrients.

[25] Tvrdý V., Hrubša M., Jirkovský E., Biedermann D., Kutý M., Valentová K., Křen V., Mladěnka P. (2021). Silymarin Dehydroflavonolignans Chelate Zinc and Partially Inhibit Alcohol Dehydrogenase. Nutrients.

[26] Wüpper S., Lüersen K., Rimbach G. (2020). Chemical Composition, Bioactivity and Safety Aspects of Kuding Tea—From Beverage to Herbal Extract. Nutrients.

[27] Yessenkyzy A., Saliev T., Zhanaliyeva M., Masoud A.-R., Umbayev B., Sergazy S., Krivykh E., Gulyayev A., Nurgozhin T. (2020). Polyphenols as Caloric-Restriction Mimetics and Autophagy Inducers in Aging Research. Nutrients.

